# Biological and Phytochemical Insights Into *Opuntia ficus‐indica* (L.) Mill: Cytotoxic, Wound‐Healing, and Anti‐Aging Potentials

**DOI:** 10.1002/fsn3.70399

**Published:** 2025-06-17

**Authors:** Gülsen Kendir, Meltem Güleç, Ayça Bal Öztürk, Gülşah Torkay, Muhammed Tilahun Muhammed, Abdullah Olgun, Ayşegül Köroğlu

**Affiliations:** ^1^ Department of Pharmaceutical Botany, Faculty of Pharmacy Süleyman Demirel University Isparta Türkiye; ^2^ Department of Pharmacognosy, Faculty of Pharmacy Istanbul University‐Cerrahpasa Istanbul Türkiye; ^3^ Department of Pharmacognosy, Faculty of Pharmacy Istinye University Istanbul Türkiye; ^4^ Department of Analytical Chemistry, Faculty of Pharmacy Istinye University Istanbul Türkiye; ^5^ Department of Stem Cell and Tissue Engineering, Institute of Health Sciences Istinye University Istanbul Türkiye; ^6^ 3D Bioprinting Design and Prototyping R&D Center Istinye University Istanbul Türkiye; ^7^ Department of Pharmaceutical Chemistry, Faculty of Pharmacy Süleyman Demirel University Isparta Türkiye; ^8^ Department of Biochemistry, Faculty of Pharmacy Istinye University Istanbul Türkiye; ^9^ Department of Pharmaceutical Botany, Faculty of Pharmacy Ankara University Ankara Türkiye

**Keywords:** anti‐aging, fruit juice, molecular modeling, *Opuntia ficus‐indica* (L.) Mill., phenolic composition, wound healing

## Abstract

*Opuntia ficus‐indica* (L.) Mill. has cultivated wild forms in many parts of the world, attracting notably with its fruit. Its fruits can be eaten raw or processed. Studies have shown that fruit juice has various pharmacological effects and significant nutritional value. This study investigated the phenolic composition of *O. ficus‐indica* fruit juice. Caffeic acid was the major component. Cytotoxic activity and wound‐healing effect were evaluated by in vitro assays. Results showed a concentration‐dependent response, with the lowest viability (111.89% ± 17.90%) at 1000 *μ*g/mL and the highest (162.25% ± 7.48%) at 250 *μ*g/mL. The extract (0–250 *μ*g/mL) had comparable wound closure to the control group. It increased the lifespan of *Caenorhabditis elegans* , an in vivo model, at 125 *μ*g/mL. The binding potential of *O. ficus‐indica* to matrix metalloproteinase 13 (MMP‐13) and glycogen synthase kinase 3 β (GSK3‐β) was investigated for its wound‐healing effect. The phytoconstituents are bound to MMP‐13 and GSK3‐β, but less than the native ligands. They bound more to MMP‐13 than to GSK3‐β. Caffeic acid showed the highest binding potential with MMP‐13 and one of the highest with GSK3‐β. This study suggests that the wound‐healing and anti‐aging effects of *O. ficus‐indica* fruit juice can be promising.

## Graphical abstract

*O. ficus‐indica* of fruit juice wound‐healing and cytotoxic effects were demonstrated. Anti‐aging effect of *O. ficus‐indica* fruit juice revealed with in vivo model. The highest binding potential with MMP‐13 was found for caffeic acid.

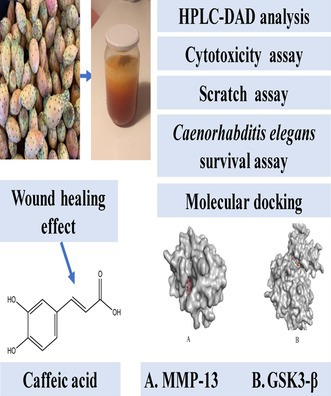

## 1 Introduction

*Opuntia ficus‐indica* (L.) Mill. is a shrub or small tree with a succulent stem, in the Cactaceae family, its branches are spread out. It is widely known around the world with names such as prickly pear and cactus pear. The plant, which is especially famous for its fruits, originates in Mexico and grows throughout a wide range of dry and semi‐arid locations on Earth, such as South America and South Africa. Both cultivated and wild forms of the plant exist in several regions of the world (Matthews 1972; Giraldo‐Silva et al. 2023; WFO 2023). The fruits, cladodes, and flowers of the plant are consumed raw or in the form of readymade products. Although the fruit is generally consumed fresh, it is also consumed in the form of processed products such as fruit juice, jam, chocolate, syrup, jelly, wine, liquor, syrup, and vinegar (Giraldo‐Silva et al. 2023; Sinicropi et al. 2022). Fruit juice is consumed at home, in restaurants, or small shops in certain nations such as Mexico and Chile (Sáenz and Sepúlveda 2001). The medical usage of prickly pears dates back a long way. Its cladodes, flowers, fruits, and seeds are used for various purposes in folk medicine. The fruits have been used in traditional medicine to treat liver and kidney inflammation, diabetes, bronchial asthma, digestive problems, wounds, diarrhea, and colitis. It is also used as an antispasmodic, astringent, emollient, and diuretic (Das et al. 2021; Kim et al. 2006). In Pakistan, it is used as a digestive booster, either raw fruit or juice (Khan and Ahmad 2015). Fruit juice is employed as a laxative and diuretic in Mexico (Brinker 2009). In Türkiye, it is known by names such as “frenk inciri, frenk yemişi, dikenli incir, kaynana dili.” The fruits are used in Türkiye as remedies for colds, anemia, intestinal pain, and kidney stones (Yeşilada 2023).

The fruit juice contains diverse bioactive components, especially flavonoids, phenolic acids, organic acids, and betalains (Galati et al. 2003; Chavez‐Santoscoy et al. 2009; Dehbi et al. 2013; Matias et al. 2014; Makhdoom et al. 2016; Mata et al. 2016; Zenteno‐Ramírez et al. 2018; Bargougui et al. 2019; Gouws et al. 2019; Palmeri et al. 2020; Martínez et al. 2021; Ferreira et al. 2022; Ferreira et al. 2023). It is a rich source of vitamin C, vitamin E, β‐carotene, and minerals (Cansino et al. 2013; Elshehy et al. 2020; Gouws et al. 2019; Zenteno‐Ramírez et al. 2018). The fruit juice displays various pharmacological effects (such as antioxidant, anticancer, antimicrobial, anticlastogenic, anti‐inflammatory, antiproliferative, antiulcerogenic, and hepatoprotective) thanks to its bioactive components (Galati et al. 2003; Galati et al. 2005; Chavez‐Santoscoy et al. 2009; Cansino et al. 2013; Dehbi et al. 2013; Madrigal‐Santillán et al. 2013; Matias et al. 2014; Bargougui et al. 2019; Gouws et al. 2019; Palmeri et al. 2020; Martínez et al. 2021; Ferreira et al. 2022; Ferreira et al. 2023). According to a clinical trial, fruit juice has substantial antioxidant activity that lowers total and LDL cholesterol, while only moderately lowering HDL cholesterol. It has also been determined that it reduces muscle damage caused by endurance exercise (Khouloud et al. 2018). In another clinical study, it was observed that fruit juice consumption after fat intake reduced autonomic cardiac regulation but did not change traditional cardiovascular disease risk responses in healthy men (Gouws et al. 2022). It has been revealed that drinking fruit juice regularly can both protect the body against oxidative stress by lowering blood sugar and delay the onset of cataract development, which is a result of diabetes (Abd El‐Razek et al. 2012). It has been shown that fruit juice can contribute to the elimination of gastric emptying disorders, as well as constipation and diarrhea in rats (Rtibi et al. 2018).

Our goal in this investigation was to ascertain the phenolic compound profile of the juice obtained by directly squeezing fresh *O. ficus‐indica* fruits and to evaluate its wound‐healing and cytotoxic effects through in vitro studies and its effect on the life span of *Caenorhabditis elegans* , an in vivo model, under heat stress. Cytotoxicity and wound‐healing effects will be demonstrated using a concentration‐dependent approach. The anti‐aging effect of *O. ficus‐indica* will be evaluated for the first time using *C. elegans* , on which a limited number of studies have been conducted. In addition, the likely mechanism of action for the wound‐healing effect observed in the biological assay was investigated by molecular docking. To the best of our knowledge, no study has examined the antiaging and wound‐healing properties of *O. ficus‐indica* fruit juice. This study will contribute to a better understanding of the therapeutic potential of *O. ficus‐indica* juice at the molecular level.

## 2 Materials and Methods

### 2.1 Materials

Dulbecco's Modified Eagle Medium (DMEM) was purchased from Capricorn Scientific GmbH (Germany). Dulbecco's phosphate‐buffered saline (DPBS) and penicillin/streptomycin (P/S) were obtained from Thermo Fisher Scientific (USA). Trypsin–EDTA was procured from Biological Industries. Dimethyl sulfoxide (DMSO), 3‐[4,5‐dimethylthiazol‐2‐yl]‐2,5‐diphenyltetrazolium bromide (MTT), and Fetal Bovine Serum (FBS) were acquired from Sigma‐Aldrich (St. Louis, MO). All chemicals and solvents employed were of analytical grade, ensuring adherence to established quality standards for research applications.

### 2.2 Plant Material and Preparation

Mature *Opuntia ficus‐indica* fruits (2.5 kg) were purchased from a local market (obtained by collecting from the surrounding area) in September 2018 in Hatay, Türkiye. The fruits were washed and peeled. Then, freshly squeezed juice was obtained directly from the peeled fruits (1.522 kg) using a juicer (Philips XXL). The resulting juices (657.613 g) were filtered and frozen in the freezer (−80°C) and then lyophilized in a Freeze Dryer (Christ Gamma 2–16 LSC, Germany).

### 2.3 HPLC–DAD Analysis

The analysis was carried out using the methodology previously outlined in Kızılyıldırım et al. (2024). Agilent Eclipse XDB C‐18 column (250 mm x 4.6 mm, 5 μm) was used in the analysis process. The injection volume was adjusted to 20 *μ*L. Two eluents that made up the mobile phase were 3% acetic acid (A) and methanol (B) in the gradient system. The rate of A was reduced from 93% to 0% in 80 min, then returned to the initial condition. Sample solutions with a concentration of 4 mg/mL were prepared. Stock standard solutions (gallic acid, protocatechic acid, caffeic acid, syringic acid, ferulic acid, rutin, cinnamic acid, quercetin, kaempferol) with a concentration of 1 mg/mL were used. Data collection and analysis were conducted using the Shimadzu Class‐VP Chromatography Laboratory Automated Software system (Shimadzu LC Solution 1.24 SP2).

### 2.4 In Vitro Cytotoxicity Studies

The cytotoxicity of *O. ficus‐indica* water‐soluble fruit juice extract was assessed using the MTT (3‐(4,5‐dimethylthiazol‐2‐yl)‐2,5‐diphenyltetrazolium bromide) assay on the NIH/3 T3 mouse embryonic fibroblast cell line (ATCC CRL‐1658) (Bal‐Ozturk et al. 2019; Yaşayan et al. 2021). A concentrated stock solution of the extract was prepared in a complete medium (DMEM (Dulbecco's Modified Eagle Medium), 10% FBS (Fetal Bovine Serum), 1% P/S (Penicillin–Streptomycin)) and sterilized through a 0.22 *μ*m polyethersulfone (PES) filter. Serial dilutions of the stock solution were prepared in a complete medium to achieve the desired testing concentrations (15.63–1000 *μ*g/mL). Cells were seeded at a density of 6 × 10^3^ per well in a 96‐well plate and incubated overnight at 37°C in a humidified 5% CO_2_ incubator. Following incubation, 100 *μ*L of complete medium containing various concentrations of the extract was added to each well, except for the negative control wells, which received fresh medium only. The plate was further incubated for 24 h under the same conditions. Subsequently, a 10% (v/v) MTT solution (5 mg/mL in phosphate‐buffered saline) was prepared in a fresh complete medium. After removing the extract‐containing and control medium, 110 *μ*L of the MTT solution was added to each well. The plates were incubated for an additional 4 h at 37°C. The MTT solution was then aspirated, and 100 *μ*L of DMSO was added to each well to dissolve the formed formazan crystals. The plates were shaken for 5 min to ensure complete homogenization. Finally, the absorbance of the dissolved formazan was measured at 570 nm using a microplate reader (BMG LABTECH SPECTROstar Nano). With the following formula, cell viability was represented as a percentage of the untreated control:

(1) $$\text{Cell Viability}\;\left(\%\right)=\frac{{\mathrm{OD}}_{570}\;\text{Sample}}{{\mathrm{OD}}_{570}\;\text{Control}}\times 100$$

where OD_570_ Sample is optical density at 570 nm of the sample, and OD_570_ Control is optical density at 570 nm of the control.

### 2.5 In Vitro Scratch Assay

The wound‐healing potential of *O. ficus‐indica* fruit juice extracts was assessed using the in vitro scratch assay on NIH/3 T3 mouse embryonic fibroblast cells (Yaşayan et al. 2021). Cells were seeded at a density of 40 × 10^3^ per well in 24‐well plates and cultured in a complete medium (DMEM, 5% FBS, 1% P/S) until reaching 90% confluency. A standardized scratch wound was created vertically across the cell monolayer using a sterilized 200 *μ*L pipette tip. Cell debris and detached cells were carefully removed by washing with DPBS (Dulbecco's phosphate‐buffered saline). Subsequently, prepared extracts (refer to MTT protocol for details) in complete medium (with 5% FBS) were added to the corresponding wells. Plates were incubated under controlled conditions at 37°C and 5% CO_2_. Wound closure was monitored and documented at predetermined time points (0, 6, and 24 h) using an inverted microscope (Observer Z1, Carl Zeiss). Images were captured, and the wound area was quantified using ImageJ software (National Institutes of Health). The percentage of wound closure was calculated using the following formula:

(2) $$\text{Wound Closure}\;\left(\%\right)=\frac{T_{0}-T_{\times }}{T_{0}}\times 100$$

where *T*_0_ is the wound area at the time of the scratch, and *T*_*x*_ is the wound area at any given time point.

### 2.6 ***Caenorhabditis elegans*** Survival Assay Under Heat Stress

Wild‐type *Caenorhabditis elegans* N2 strain was obtained from the Caenorhabditis Genetics Center (CGC) at the University of Minnesota. Nematodes were cultivated and maintained on nematode growth medium (NGM) containing plates seeded with *Escherichia coli* OP50‐1, as described by Stiernagle (2006), at 22°C ± 2°C. For thermotolerance assays, bacteria were heat‐inactivated at 65°C for 10 min. Only synchronized, healthy, and uncontaminated populations of worms were used. The study conditions are given in Table S1.

After being placed on fresh NGM agar plates, eggs were allowed to hatch and reach the stage of young adulthood before being moved to the experimental plates. For the experimental groups, extracts at different concentrations were added to the L broth that also contained *E. coli* . Three plates were prepared for each group (Alparslan et al. 2023).

The worms were kept in prepared petri dishes at room temperature for 24 h. They were then moved to a 35°C incubator (Lithgow et al. 1994). Until all of the worms died, plates' pictures were taken every 20 min using an Epson Perfection V800 Photo high‐resolution scanner. Dead worms were defined as those that stayed motionless for two consecutive scans.

Survival data were analyzed using the Kaplan–Meier method. Statistical significance between groups was assessed with the log‐rank (Mantel–Cox) test. Bonferroni correction was applied to adjust for multiple comparisons. Additionally, Fisher's exact test was used to evaluate differences at specific mortality thresholds (25%, 50%, 75%, and 90%). Analyses were conducted using the OASIS online survival analysis tool (Yang et al. 2011). A *p*‐value < 0.05 before correction was considered statistically significant.

### 2.7 Molecular Docking

The molecular docking of the phytoconstituents on the target enzymes was performed through AutoDock Vina (Trott and Olson 2010). The crystal structures of the target enzymes were retrieved from the protein data bank (PDB). The structure of matrix metalloproteinase 13 (MMP‐13) with a PDB code of 5UWK had a co‐crystallized ligand, (S)‐3‐methyl‐2‐(4′‐(((4‐oxo‐4,5,6,7‐tetrahydro‐3H‐cyclopenta[d]pyrimidin‐2‐yl)thio)methyl)‐[1,1′‐biphenyl]‐4‐ylsulfonamido)butanoic acid (Choi et al. 2017). Similarly, the structure of glycogen synthase kinase 3 β (GSK3‐β) with a PDB code of 8AV1 had a co‐crystallized ligand, 2‐pyridin‐3‐yl‐8‐thiomorpholin‐4‐yl‐[1,3]oxazolo[5,4‐f]quinoxaline (Hasyeoui et al. 2023). The MMP‐13 and GSK3‐β structures utilized had resolutions of 1.60 Å and 2.15 Å, respectively. The compound structures were obtained from PubChem. The enzymes and compounds were made ready for docking as described in reported studies (Erdoğan et al. 2024; Muhammed et al. 2023). The docking results were visualized through Biovia Discovery Studio Visualizer 2021. The docking protocol was validated by redocking the native ligands on the respective structures.

### 2.8 Statistical Analysis

GraphPad Prism Software (V.8.4, San Diego, USA) including one‐way ANOVA, was used for statistical analysis as mean ± standard deviation (SD). Comparisons were performed according to the Tukey test. A *p*‐value of < 0.05 was considered statistically significant.

## 3 Results and Discussion

### 3.1 Chromatographic Profile of Bioactive Compounds

**FIGURE 1:**
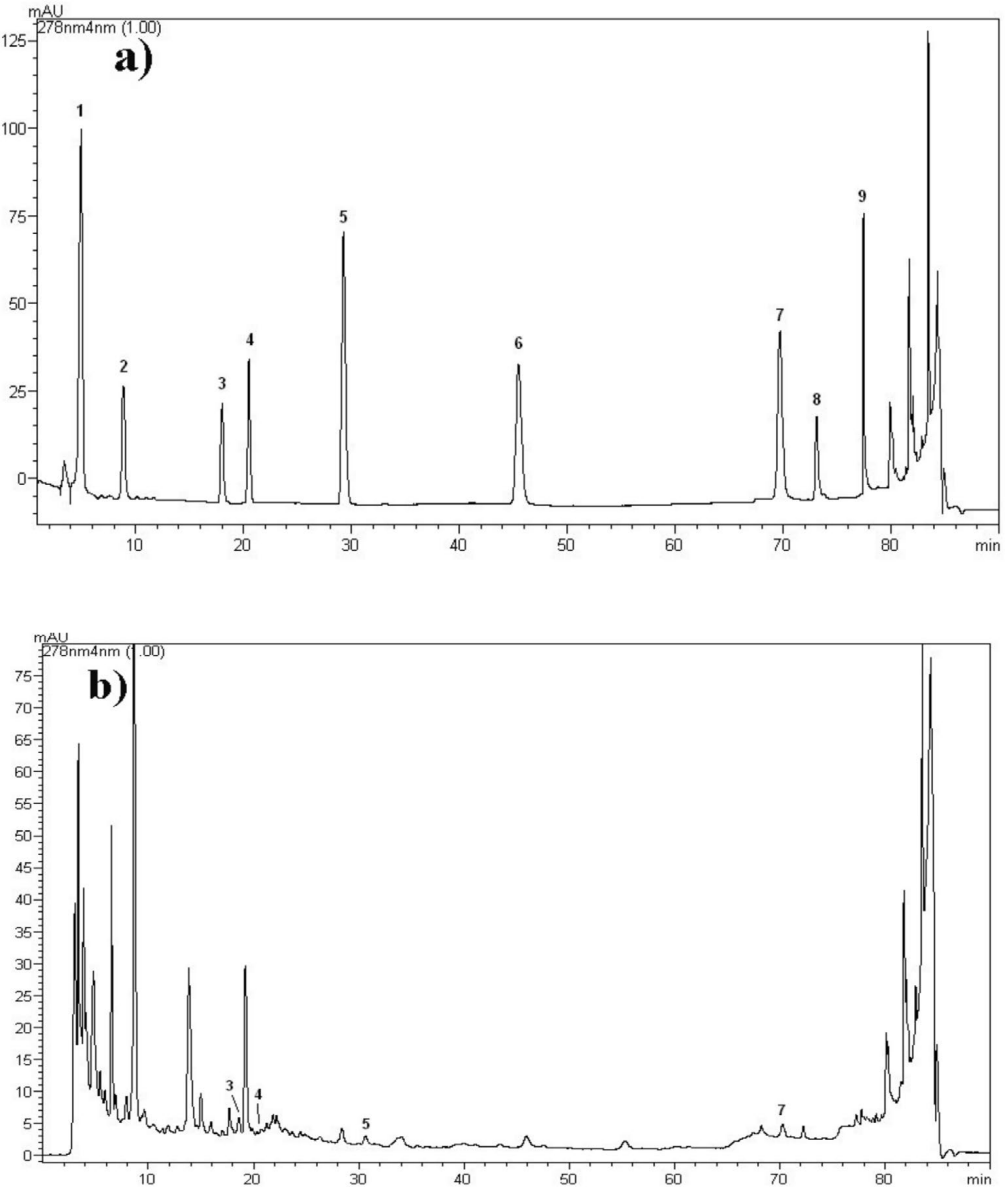
(**a)** HPLC chromatogram of the standard mixture, (**b)** HPLC chromatogram of the *Opuntia ficus‐indica* fruit juice. 1: Gallic acid; 2: Protocatechuic acid; 3: Caffeic acid; 4: Syringic acid; 5: Ferulic acid; 6: Rutin; 7: Cinnamic acid; 8: Quercetin; 9: Kaempferol.

The fruit juice yield (%) was found to be 11.5%. The contents of bioactive compounds (gallic acid, protocatechuic acid, caffeic acid, syringic acid, ferulic acid, rutin, cinnamic acid, quercetin, and kaempferol) were analyzed in the fruit juice by HPLC analysis method. HPLC chromatograms of the standard mixture and the bioactive compounds are presented in Figures 1a,b, respectively. Calibration values for the standards are given in Table S2. Gallic acid, protocatechuic acid, rutin, quercetin, and kaempferol were not detected in the fruit juice. The amounts of caffeic acid (18.6 min), ferulic acid (29.6 min), syringic acid (20.2 min), and cinnamic acid (70.2 min) were detected as 10.8668 ± 0.1020 *μ*g/g, 9.57 ± 0.2346 *μ*g/g, 8.6365 ± 0.2723 *μ*g/g, and 7.5626 ± 0.1392 *μ*g/g, respectively. Caffeic acid was observed as a major compound in *O. ficus‐indica* fresh fruit juice (Table 1).

**TABLE 1:** Content of phenolic compounds in *O. ficus‐indica* (L.) Mill. fruit juice.

Compound	Concentrations (μg/g ± SD)	Retention times (min)	Molecular formulas
Caffeic acid	10.8668 ± 0.1020	18.6	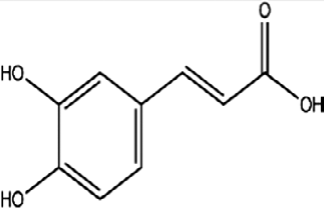
Syringic acid	8.6365 ± 0.2723	20.2	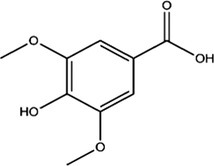
Ferulic acid	9.57 ± 0.2346	29.6	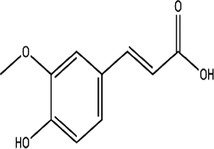
Cinnamic acid	7.5626 ± 0.1392	70.2	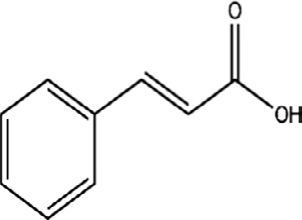

Abbreviation: SD, Standard Deviation.

*O. ficus‐indica* fruits have very rich nutritional values in terms of vitamins and minerals and are used for various purposes in traditional medicine in different parts of the world. Studies have revealed many pharmacological activities of fruits and fruit juices based on traditional use. In this study, we investigated the phenolic composition of the fruit juice by HPLC DAD analysis. Some of the main chemical compounds whose existence has been proven by previous studies were selected for analysis (Bargougui et al. 2019; Galati et al. 2003; Makhdoom et al. 2016; Martínez et al. 2021; Mata et al. 2016; Zenteno‐Ramírez et al. 2018). With the analysis, the presence of gallic acid, protocatechuic acid, rutin, quercetin, and kaempferol could not be determined. In a study conducted in Italy, it was stated that the presence of ferulic acid was observed as the main representative of hydroxycinnamic acids, although it was present in small amounts. Also, small amounts of rutin were determined (Galati et al. 2003). Caffeic acid was detected as a major component (Table 1, Figure 1b). Song et al. reported that caffeic acid displayed strong antioxidant and anti‐inflammatory effects in the cell culture system, and this may be associated with wound healing in skin‐incised mice (Song et al. 2008). In Saudi Arabia, the phenolic compounds of fruit juice were analyzed before and after enzymatic treatment, and it was observed that the amount of phenolic compounds generally increased with the enzymatic process. In this study, the amounts of gallic acid and protocatechuic acid were also determined (Makhdoom et al. 2016). The presence of rutin was detected in fruit juice obtained from wild *O. ficus‐indica* grown in Portugal (Mata et al. 2016). In a study conducted in Mexico, the amount of gallic acid was determined. The amount of syringic acid (29.2 *μ*g/g) was determined to be higher than our finding (Zenteno‐Ramírez et al. 2018). Similar to our study, caffeic acid was determined to be the main phenolic compound in the ethyl acetate extract of the fruit juice of the Ain Amara cultivar grown in Tunisia (Bargougui et al. 2019). Another study conducted in Mexico determined high amounts of gallic acid (21.75 ± 0.75 mg/L fresh sample) in the fruit juice unlike our study (Martínez et al. 2021). The profile and amount of phenolic compounds of plants vary especially with the growing conditions and vegetation period of the plant. In addition, the extraction and analysis conditions of phenolic compounds also affect the profile and amount of phenolic compounds (Cartea et al. 2010).

### 3.2 In Vitro Cytotoxicity Results

**FIGURE 2:**
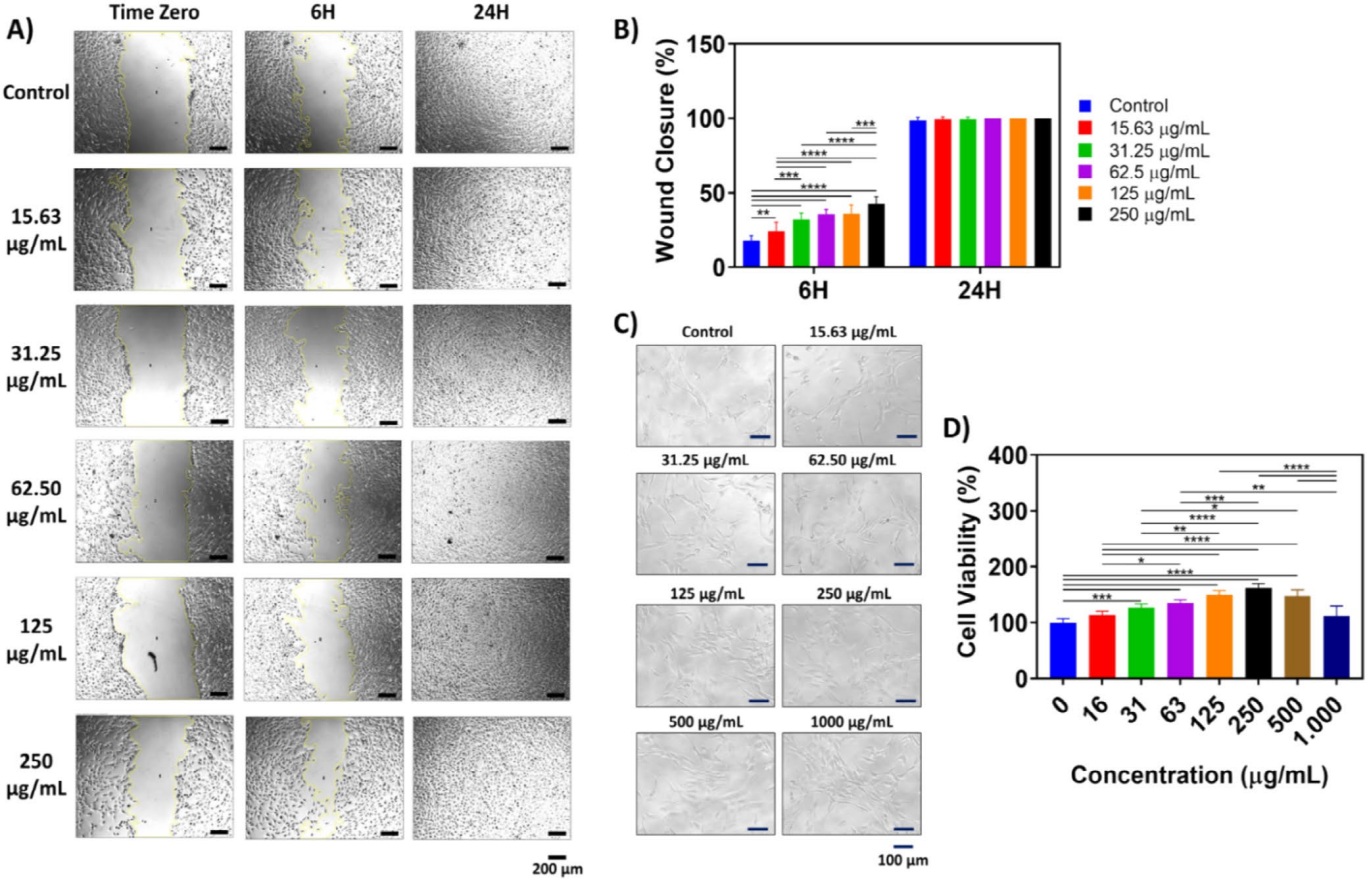
Cell culture study results of the *Opuntia ficus‐indica* fruit juice: (A) Representative images of in vitro scratch testing, scale bar: 200 *μ*m, (B) In vitro scratch test results, (C) Morphologies of fibroblast cells after 24 h of treatment, scale bar: 100 *μ*m, (D) MTT test results (Data show the mean ± SD, *n* = 6 for the MTT results and *n* = 8 for the scratch results, one‐way ANOVA with Tukey's post hoc test, ns: *P* > 0.05, *p < 0.05, **p < 0.01, ***p < 0.001.

Given the growing interest in the diverse bioactive potential of *O. ficus‐indica* , particularly its fruit extract rich in phenolic acids and flavonoids, the cytotoxicity of *O. ficus‐indica* fruit juice was assessed using the MTT assay on the NIH/3 T3 mouse embryonic fibroblast cells. Cells were treated with varying concentrations of the fruit juice extract for 24 h, and their viability was compared to an untreated control (Figure 2D). A concentration‐dependent response was observed, with the highest viability (162.25 ± 7.48%) at 250 *μ*g/mL and the lowest (111.89 ± 17.90%) at 1000 *μ*g/mL. Statistical analysis revealed no significant difference in viability compared to the control at 15.63 and 1000 *μ*g/mL (*p* > 0.05). However, significant differences were observed such as high significance (*p* ≤ 0.001) at 31.25 *μ*g/mL and very high significance (*p* ≤ 0.0001) at all other concentrations. Cell morphology remained unaffected by increasing viability, as shown in Figure 2C. These results suggest that the *O. ficus‐indica* fruit juice extract can be used safely in various biological applications.

Prior studies on *O. ficus‐indica* have primarily examined its anticancer properties, highlighting concerns regarding possible cytotoxic effects on noncancerous cells (Ali et al. 2022; Becer et al. 2018; Sharma et al. 2022; Sharma et al. 2023). Additionally, its potential applications in wound dressings, topical formulations, and other areas of biomedicine required cytotoxicity evaluation. In alignment with previous research, our study showed that the fruit juice extract did not exhibit cytotoxic effects on NIH/3 T3 fibroblasts within the examined concentration range. A notable increase in cell viability was observed, especially at a concentration of 250 *μ*g/mL, indicating a potential proliferative effect. The observed phenomenon can be assigned to the antioxidant and cytoprotective properties of caffeic acid, the main phenolic compound present in the extract. Research indicates that low levels of caffeic acid may improve cell viability and offer protection against oxidative stress‐related damage, possibly through the activation of the ERK signaling pathway (Li et al. 2015; Ul Haq et al. 2023). A study by Martínez et al. (2021) confirmed previous findings, demonstrating that purified juice from yellow *O. ficus‐indica* fruit selectively targeted liver cancer cells while leaving normal fibroblasts unaffected. The findings further support the biocompatibility of *O. ficus‐indica* fruit juice and offer additional evidence for its potential integration in bioactive wound care formulations.

### 3.3 In Vitro Scratch Assay Results

The influence of *O. ficus‐indica* fruit juice extract on fibroblast migration and proliferation was evaluated using the in vitro scratch assay across a concentration range of 0–250 *μ*g/mL. At the 6‐h time point, a concentration‐dependent increase in wound closure was observed (Figures 2A,B), suggesting an early‐stage stimulation of fibroblast migration and/or proliferation. Notably, by the 24‐h mark, all extract‐treated groups exhibited similar wound closure to the control group, indicating no inhibitory effect on cellular migration and supporting the biocompatibility at later stages. It was concluded that *O. ficus‐indica* fruit juice extract can be formulated for use in wound dressing applications.

Fibroblasts, crucial contributors to the extracellular matrix, are commonly employed in such studies. The wound‐healing activity of *O. ficus‐indica* stems has been demonstrated in an in vivo study (Park and Chun 2001). It is noted that in an excision wound model in rats, the self‐nanoemulsifying drug delivery formula of *O. ficus‐indica* seed oil exhibited a greater wound‐healing activity than conventional *O. ficus‐indica* seed oil (Koshak et al. 2021). The short‐term enhancement can be partly explained by the action of caffeic acid, the predominant phenolic constituent of the extract. Caffeic acid derivatives were also previously reported to enhance wound repair by stimulating cell migration, proliferation, and angiogenic response in conditions of oxidative or hypoxia stress (Park et al. 2022). To our knowledge, there has been no comparison of the direct effect of fresh fruit juice on in vitro fibroblast migration so far. Therefore, the present study gives the first evidence in support of the use of *O. ficus‐indica* fruit juice in wound‐healing cases.

### 3.4 ***Caenorhabditis elegans*** Survival Assay Under Heat Stress

**FIGURE 3:**
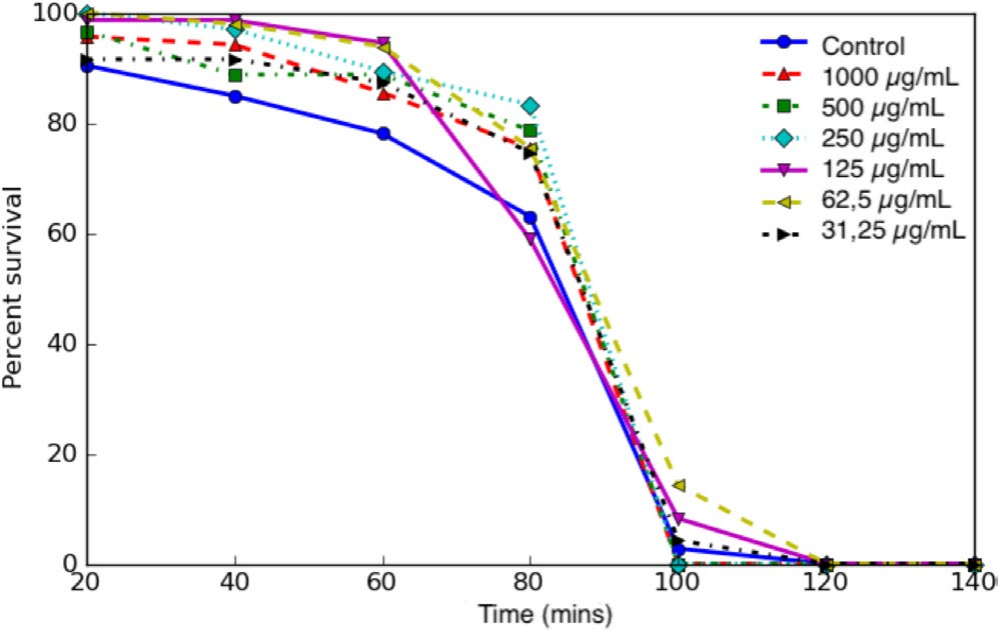
Survival curve of *C. elegans* treated with *Opuntia ficus‐indica* fruit juice.

In the presence of heat stress, the lifespan of *C. elegans* was assessed at varying concentrations of the extract. The Kaplan–Meier survival curves indicated that specific extract concentrations resulted in a slight improvement in lifespan when compared with the control group (Figure 3).

While unadjusted *p*‐values showed significant differences for Group 3 (250 *μ*g/mL, *p* = 0.0222) and Group 5 (62.5 *μ*g/mL, *p* = 0.0143) compared with the control, these differences were not statistically significant after Bonferroni correction. Group 2 (500 *μ*g/mL) also showed a trend toward significance (*p* = 0.0701), though this was not maintained postcorrection (Table 2).

**TABLE 2:** Log‐rank test results comparing extract‐treated groups with control.

Groups	Chi^2^	*p*‐value	Bonferroni‐corrected
Group 1 vs. Control	1.6	0.2056	1.0000
Group 2 vs. Control	3.28	0.0701	0.4204
Group 3 vs. Control	5.23	0.0222	0.1331
Group 4 vs. Control	8.96	0.0028	1.0000
Group 5 vs. Control	6.01	0.0143	0.0855
Group 6 vs. Control	2.44	0.1183	0.7098

Group 3 (250 *μ*g/mL) showed a significant difference compared with the control group at 25% mortality (*p* = 0.0080), while Group 5 (62.5 *μ*g/mL) showed significance at higher mortality thresholds (*p* = 0.0293). A consistent and highly significant difference was observed between Group 2 and Group 5 across all thresholds (*p* = 0.0005).

The mean lifespan of Group 5 was the longest among all experimental groups, reaching 96.4 min, compared to 94 min in Group 3 and shorter durations in other groups (Table 3).

**TABLE 3:** Fisher's exact test *p*‐values at different mortality thresholds.

Groups	*p*‐value at 25%	*p*‐value at 50%	*p*‐value at 75%	*p*‐value at 90%
Group 1 vs. Control	0.1464	0.4969	0.4969	0.4969
Group 2 vs. Control	**0.0356**	0.2015	0.2015	0.2015
Group 3 vs. Control	**0.0080**	0.4977	0.4977	0.4977
Group 4 vs. Control	0.7346	0.2752	0.2752	0.2752
Group 5 vs. Control	0.1693	**0.0293**	**0.0293**	**0.0293**
Group 6 vs. Control	0.1525	0.6787	0.6787	0.6787
Group 2 vs. Group 5	**0.0005**	**0.0005**	**0.0005**	**0.0005**

For the first time, the fruit juice study was conducted. It was reported that the lifespan of *C. elegans* was shown to be significantly extended, with a maximum lifespan of up to 14.2 days and an average survival rate of 21.06% when yellow *O. ficus‐indica* fruit extract was used at a 0.5% w/v concentration (Guerrero‐Rubio et al. 2019). Our study demonstrates a direct correlation between concentration levels and lifespan, with higher concentrations (1000 *μ*g/mL, 500 *μ*g/mL, and 250 *μ*g/mL) resulting in significant improvements in lifespan compared to the control group. The observed increases ranged from approximately 12% to 18%.

### 3.5 Molecular Docking

**FIGURE 4:**
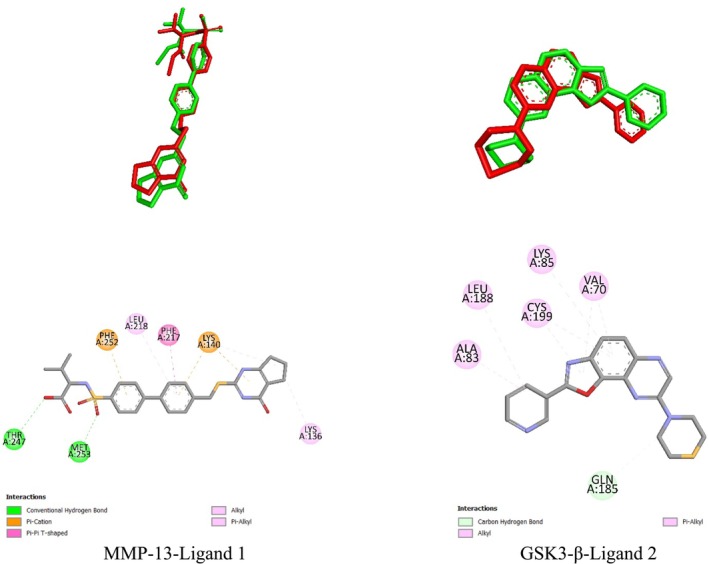
Superimposed redocked (red) and crystal (green) structures of the native ligands together with their interactions with the corresponding target structures.

The biological assay investigation showed that the fruit extract had a wound‐healing effect. Hence, the probable mode of action for the detected wound‐healing activity was explored by using suitable targets involved in the wound‐healing process. To this end, the binding potential of the phytoconstituents of *O. ficus‐indica* fruit juice with MMP‐13 and GSK3‐β was investigated through molecular docking. Before proceeding to the docking of the phytoconstituents, the docking procedure to be employed was validated by redocking the co‐crystallized ligands to the target enzyme structures. Then, the RMSD values between the redocked and the crystal structures were computed. The RMSD value between the redocked native ligand embedded in the MMP‐13 crystal structure (ligand 1) and its crystal structure was found to be 0.8276 Å. Similarly, the RMSD value between the redocked native ligand embedded in the GSK3‐β crystal structure (ligand 2) and its crystal structure was found to be 1.7869 Å (Figure 4). Ligand 1 interacted with the MMP‐13 structure (5UWK) through three conventional hydrogen bonds and seven other types of interactions with a binding affinity of −8.2 kcal/mol (Figure 4, Table 4). On the other hand, ligand 2 did not form a conventional hydrogen bond with GSK3‐β. It formed just eight other types of interactions. Together with this, the binding affinity of ligand 2 to the enzyme would be high as it was found to be −8.6 kcal/mol.

**TABLE 4:** Binding affinity and bonding types of the phytoconstituents in their interactions with MMP‐13 (5UWK) and GSK3‐β (8AV1).

Compound	Target	Binding affinity (kcal/mol)	Inhibition constant (Ki in μM)	Conventional hydrogen bonding residues	Other interaction residues
Caffeic acid	5UWK	−7.4	3.76	Phe241, Ile243	Val219^a^, His222^b^, His222^c^, Tyr244^b^
Cinnamic acid	5UWK	−6.7	12.26	Thr245	Val219^a^, His222^b^, His222^c^, Tyr244^b^
Ferulic acid	5UWK	−7.2	5.27	Ile243, Thr245	Val219^a^, His222^a^, His222^b^, His222^c^, His232^a^
Syringic acid	5UWK	−6.0	39.5	Phe241, Ile243, Thr245	Leu218^a^, Leu218^d^, His222^e^, Phe241^e^, Tyr244^e^, Thr245^f^
Ligand 1	5UWK	−8.2	0.97	Thr247, Met253 (2)	Lys136^e^, Lys140^e^, Lys140(2)^c^, Phe217^b^, Leu218^a^, Phe252^c^
Caffeic acid	8AV1	−5.9	47.3	Val135, Asp200	Val70^a^, Ala83^a^, Lys85^a^, Leu132^a^, Cys199^g^
Cinnamic acid	8AV1	−5.5	92.91	Tyr134	Ala83^a^, Leu188^h^, Cys199^a^
Ferulic acid	8AV1	−5.9	47.3	Tyr134, Val135	Val70^e^, Ala83^a^, Leu188^h^, Cys199^a^
Syringic acid	8AV1	−5.6	78.48	Asp200, Phe201	Phe67^e^, Val70^e^, Ala83^e^, Lys85(2)^a^, Val110^e^, Leu132^h^, Leu132^e^, Asp133^d^, Leu188^e^, Cys199^a^
Ligand 2	8AV1	−8.6	0.50	—	Val70(2)^a^, Ala83^a^, Lys85^d^, Gln185^a^, Leu188^a^, Cys199(2)^a^

^a^ pi‐alkyl.

^b^ pi‐pi.

^c^ pi‐ion.

^d^ carbon hydrogen bond.

^e^ alkyl.

^f^ unfavorable donor–donor.

^g^ pi‐sulfur.

^h^ pi‐sigma.

**FIGURE 5:**
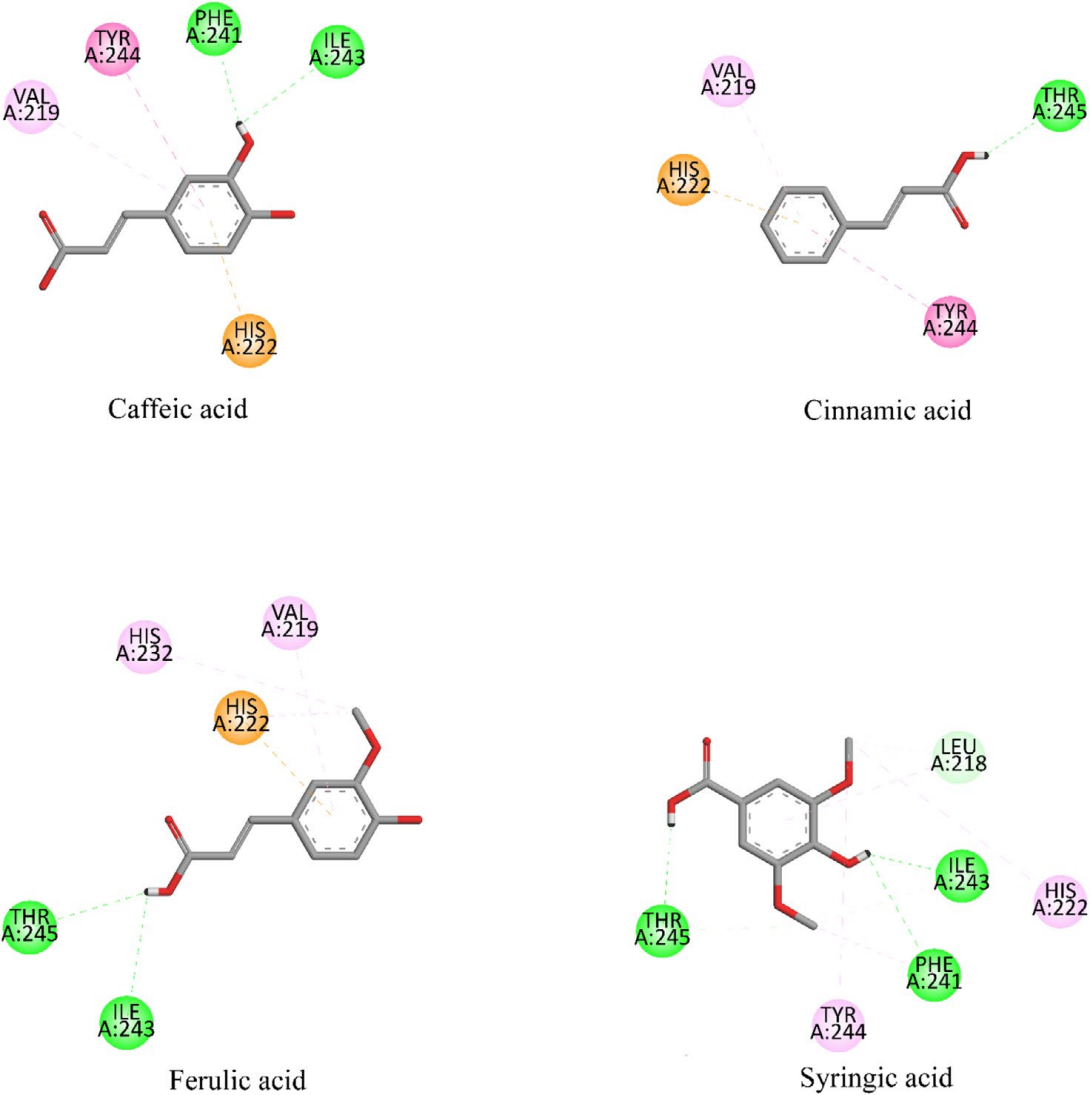
Binding residues and bonding types of caffeic acid, cinnamic acid, ferulic acid, and syringic acid in their interaction with MMP‐13 (5UWK). In the figure the color representation is as: Green (conventional hydrogen bond), lemon (carbon hydrogen bond), pink (pi‐pi), pale pink (pi‐alkyl/alkyl), and orange (pi‐ion).

The biochemical analysis of *O. ficus‐indica* fruit juice demonstrated that caffeic acid, cinnamic acid, ferulic acid, and syringic acid are the phytoconstituents of the extract. The binding potential of the phytoconstituents with MMP‐13 (5UWK) was explored through docking. The binding affinities of the phytoconstituents were found to be lower than that of the native ligand (ligand 1). The highest binding affinity toward MMP‐13 was shown by caffeic acid. The binding affinity of caffeic acid (−7.4 kcal/mol) was still lower than the binding affinity of ligand 1 (−8.2 kcal/mol). The other phytoconstituents had a lower binding affinity toward MMP‐13. With this being noted, the binding affinity difference among the phytoconstituents was not big (Table 4). The binding strength achieved by the interaction of the phytoconstituents would also be lower than that of ligand 1. Ligand 1 formed a stronger interaction via three conventional hydrogen bonds and seven other types of interactions (Table 4, Figure 5). Syringic acid had three conventional hydrogen bonds with MMP‐13. However, it had an unfavorable donor–donor interaction at the same time. This would weaken the interaction strength of syringic acid with the enzyme. The rest of the phytoconstituents had a lower number of conventional hydrogen bonds and other interactions with the enzyme (Figure 5, Table 4). Therefore, the phytoconstituents are anticipated to exhibit a lower binding potential to MMP‐13 relative to the native ligand.

**FIGURE 6:**
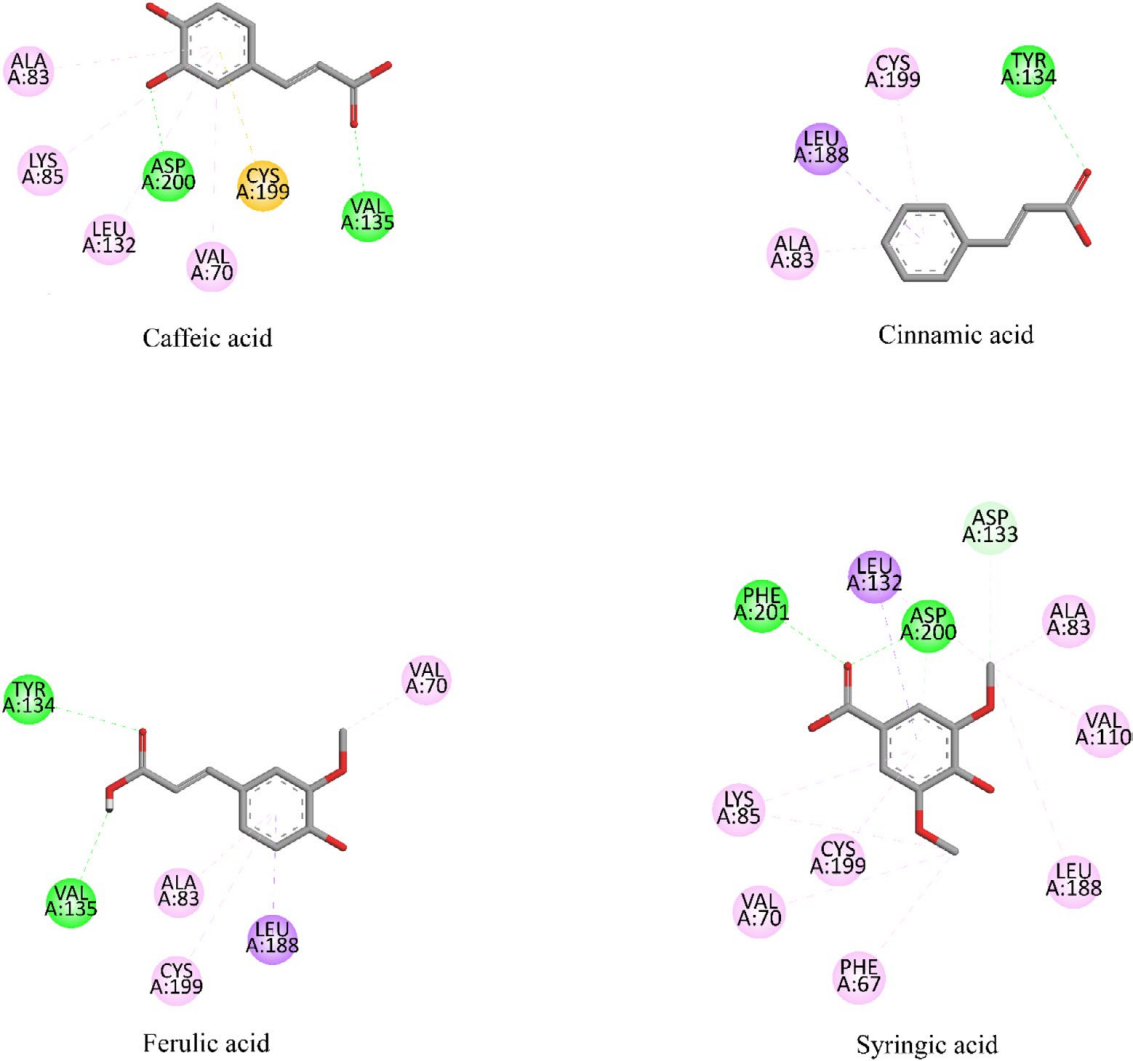
Binding residues and bonding types of caffeic acid, cinnamic acid, ferulic acid, and syringic acid in their interaction with GSK3 β (8AV1). In the figure the color representation is as: Green (conventional hydrogen bond), lemon (carbon hydrogen bond), pink (pi‐pi), pale pink (pi‐alkyl/alkyl) purple (pi‐sigma), and orange (pi‐ion).

The binding potentials of the phytoconstituents of the extract to GSK3 β (8AV1) were investigated through docking. The docking results showed that the native ligand (ligand 2) had a higher binding affinity to the enzyme relative to the phytoconstituents. Among the phytoconstituents, caffeic acid (−5.9 kcal/mol) and ferulic acid (−5.9 kcal/mol) had relatively higher binding affinities than cinnamic acid (−5.5 kcal/mol) and syringic acid (−5.6 kcal/mol). The difference among the binding affinities of the phytoconstituents of the extract was lower than the difference with ligand 2 (Table 4). On the other hand, the binding strength of the phytoconstituents was found to be slightly higher than that of ligand 2 because the phytoconstituents formed conventional hydrogen bonds with the enzyme (Figure 6, Table 4). The interaction of cinnamic acid had a conventional hydrogen bond. However, the number of other types of interactions was low (three). Caffeic acid and ferulic acid formed two conventional hydrogen bonds. The number of other types of interactions formed by caffeic acid and ferulic acid was five and four, respectively. Ligand 2 formed eight other types of interactions with the enzyme (Table 4, Figure 6). The higher number of other interactions by the native ligand is anticipated to compensate for the conventional hydrogen bond difference to some level. As a result, the interaction strength difference between the native ligand and the two components would not be that much. However, syringic acid formed eleven other types of interactions in addition to the two conventional hydrogen bonds. As a result, syringic acid is expected to form a stronger interaction with the enzyme relative to the native ligand. Together with this, all of the phytoconstituents had lower binding affinities to the enzyme relative to the native ligand.

The possible mode of action for the detected wound‐healing activity of the extract was explored through docking. The docking of the phytoconstituents with MMP‐13 and GSK3‐β was undertaken for this purpose. Matrix metalloproteinases (MMPs) clear damaged extracellular matrix from the wound. MMP‐13 (collagenase 3) is a member of mammalian MMPs that play a crucial role in tissue rebuilding and repair. Researchers also reported its role in the progression of some diseases, including cancer, arthritis, and atherosclerosis (Stura et al. 2013). Especially, its involvement in cancer progression led us to select it as a target. The cytotoxic activity of the extract was also investigated in the biological assay. Targeting MMP‐13 will also serve to explain the possible anticancer effect of the extract.

The docking procedure was validated by redocking the native ligands on the target structures. The RMSD value of redocked ligand 1 and its crystal structure was found to be 0.8276 Å. The RMSD value was much lower than the threshold value for reliable docking (2 Å) (Muhammed and Aki‐Yalcin 2024). Thereafter, the interactions of the native ligand with the enzyme were compared to the reported crystallographic and computational studies. A crystallographic study reported the interaction of some ligands with the enzyme via Thr247 and Phe252 (Choi et al. 2017). Another crystallographic study consisting of a ligand reported interaction via Leu218 (Stura et al. 2013). These interactions obtained from the experimental studies were observed in the computational study (Table 4, Figure 4). A computational study reported the interaction of a ligand with the enzyme via Leu218 and Thr247 (Cai et al. 2020). Another computation consisting of a study reported the interaction of a compound with the enzyme via Thr247 and Phe252 as observed in the computational study (Table 4, Figure 4) (Fuerst et al. 2022). Some level of similarity between the interaction residues of the native ligand in this study and previous studies was achieved. The low RMSD value between the redocked and crystal native ligand indicated a reliable docking procedure. The two structures are supposed to settle in a similar vicinity to the enzyme's binding site. The medium level of similarity in amino acid interaction residues of the native ligand in its binding with the enzyme to the reported studies supported the high similarity of settlement for the two structures.

The interaction residues of the phytoconstituents in their interaction with MMP‐13 had high similarity to the reported experimental and computational studies. In this regard, previous experimental studies reported the interactions of various compounds with the enzyme via Leu218, Phe241, Ile243, Tyr244, and Thr245 as observed in the interaction of the phytoconstituents (Choi et al. 2017; Stura et al. 2013). A computational study reported the interaction of a ligand to MMP‐13 via various residues, including His222, His232, and Tyr244 (Bikádi et al. 2006). Another computational study reported the interaction of a compound to the enzyme via Leu218, Ile243, Tyr244, and Thr245 residues (Cai et al. 2020). Similarly, a computational study reported the interaction of a compound with the enzyme via His222, His232, and Thr245 residues (Fuerst et al. 2022). Another computational study reported the interaction of a compound with the enzyme via Val219, Ile243, and Tyr244 residues (Duan et al. 2023). The interactions observed in this study were found to be highly coherent with the reported computational studies. In short, the docking results of the computational study were highly compatible with the reported experimental and computational studies, as all of the interaction residues were observed in earlier studies. In the docking study, the interaction of the phytoconstituents through His222 was found to be critical in their interaction with the enzyme. This result was also in line with the reported computational studies, as two previously reported studies showed the interactions of different molecules via His222 (Bikádi et al. 2006; Fuerst et al. 2022). As a result, it is possible to infer that the phytoconstituents could bind to the enzyme but with less potential than the native ligand. Furthermore, caffeic acid is anticipated to have the highest potential to bind to the enzyme (Figure 4, Table 4).

The interaction potential of the phytoconstituents with GSK3‐β was investigated through molecular docking. GSK3‐β is a member of the serine/threonine kinase family that is known to be involved in the Wnt‐beta catenin pathway (Paramesha et al. 2015). Activation of GSK3‐β delays the wound‐healing process. Hence, inhibition of GSK3‐β is an important strategy to promote wound healing (Subbukutti et al. 2024). As a result, the inhibition potential of the phytocomponents against GSK3‐β was explored through docking. Before proceeding to the docking of the phytoconstituents, the docking procedure was validated by redocking the native ligand in the crystal structure used. The RMSD value between the redocked and crystal native ligand structure was found to be 1.7869 Å. This value is below the upper threshold for reliable docking (2 Å) (Muhammed and Aki‐Yalcin 2024). Hence, the docking protocol of the GSK3‐β(8AV1) met this requirement. Thereafter, the binding residue similarity of the native ligand (ligand 2) was compared to the available experimental and computational study literature with the target enzyme. The interaction through Lys85 was a common residue with the crystallographic study that reported the GSK3‐β structure utilized in this study. A computational study reported some residues as connection points or stable residues. The list included Val70, Ala83, Lys85, Gln185, Leu188, and Cys199, which are the residues of interaction for the native ligand in this study (Zareei et al. 2024). Another computational study revealed the interaction of various ligands with the enzyme via Val70, Ala83, Lys85, Leu188, and Cys199 as the native ligand (Subbukutti et al. 2024). Similarly, another computational study reported the interaction of phytocomponents with the enzyme via residues including Val70, Ala83, Lys85, Leu188, and Cys199 as the native ligand (Aksoy et al. 2021). Therefore, a high level of similarity between the reported computational studies and the interaction residues of the native ligand in this study was observed. The high level of similarity with reported studies, especially with computational studies, enhanced the reliability of the docking procedure that gave an acceptable RMSD result.

**FIGURE 7:**
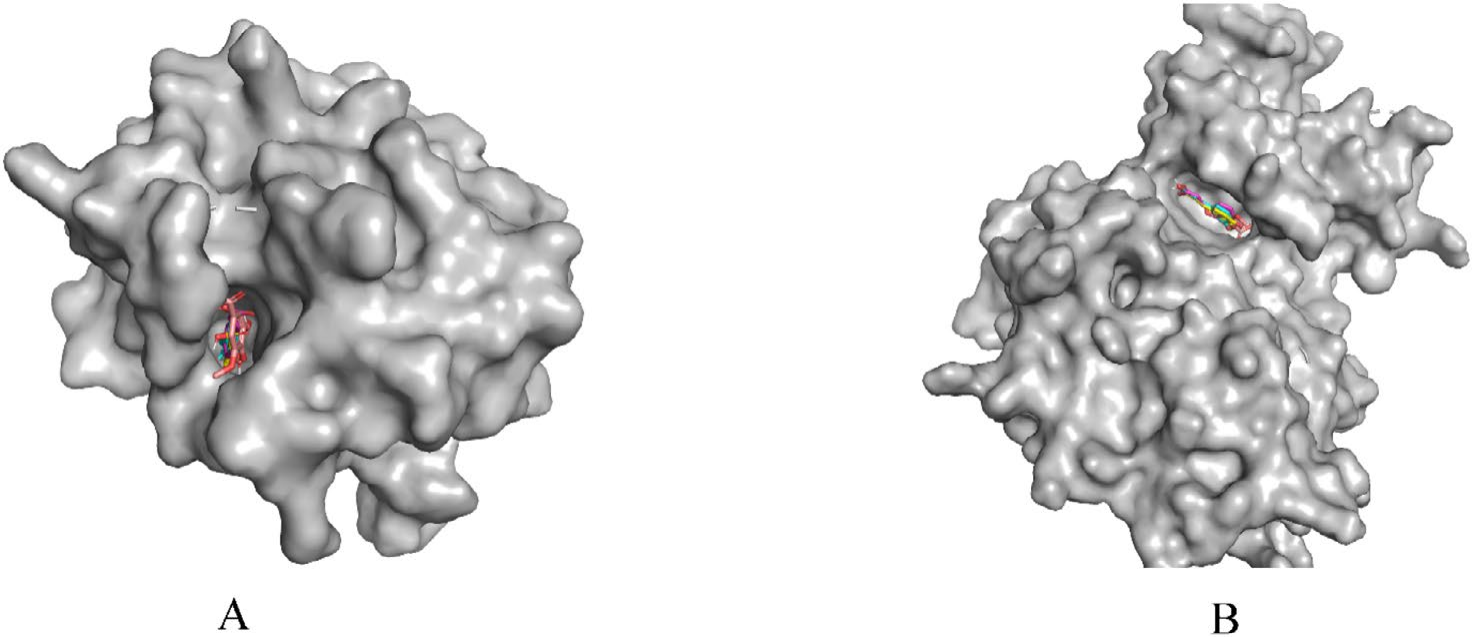
Orientation of the major phytocomponents inside the binding sites of the 5UWK‐coded MMP‐13 structure (A) and 8AV1‐coded GSK3 β structure (B).

The interactions of the phytoconstituents with GSK3‐β were similar to the interactions of the native ligand and the reported computational studies. All the interactions of the native ligand with the enzyme, except the one via Gln185, were observed in the interactions of the phytoconstituents. The interaction residues that were different from the residues of the native ligand were also observed in reported computational studies. In this regard, a computational study reported interactions of ligands with the enzyme via Asp133, Val135, and Asp200 (Zareei et al. 2024). Similarly, another computational study reported the interaction of various ligands to the enzyme via various residues, including Val110, Leu132, Tyr134, Val135, and Asp200 as observed in this study (Subbukutti et al. 2024). Another computational study also reported the interaction of some phytocomponents with the enzyme via Val110, Leu132, Asp133, Tyr134, Val135, and Asp200 residues (Aksoy et al. 2021). A computational analysis containing a study reported the interaction of two compounds with the enzyme via Phe67 and Tyr134 (Zhu et al. 2020). An interaction of ligands to the enzyme via Phe67 and Asp200 was also reported by a previous computational study (Quesada‐Romero and Caballero 2014). Hence, all of the interactions of the phytoconstituents to the enzyme, except the one via Phe201, were observed in the available literature supported by docking. In the docking study, the phytoconstituents were found to exhibit a lower binding affinity toward GSK3‐β relative to the native ligand. Together with this, the phytoconstituents had a comparable level of interaction strength with the native ligand. Caffeic acid was found to show the highest binding affinity with a considerable binding strength relative to the rest of the phytoconstituents (Table 4, Figure 6). The inhibition constant of the major phytocomponents in their binding to the two enzymes was computed. The inhibition constant was calculated by taking the exponential of the change in binding energy divided by the gas constant (1.987 cal/K.mol) and temperature (298.15 K). Caffeic acid was the compound that gave the lowest inhibition constant in its interaction to the two enzyme structures (Table 4). This has shown that caffeic acid would have a higher contribution to the binding and thus inhibition of the extract to the target enzymes. Ferulic acid also gave one of the lowest inhibition constants in the interaction to GSK3‐β implying its contribution to the binding (Table 4). To wrap up, the docking study revealed that the phytoconstituents had lower binding potential to MMP‐13 and GSK3‐β relative to the native ligands. Major phytocomponents were found to be submerged inside the binding regions of the respective target structures (Figure 7). The phytoconstituents had relatively higher binding potential toward MMP‐13. Among the phytoconstituents, caffeic acid exhibited the highest binding potential toward the two target enzymes.

The data obtained in the study largely coincide with the objectives determined at the beginning of the research. The phytochemical analysis successfully identified caffeic acid as the major phenolic compound in *Opuntia ficus‐indica* fruit juice, fulfilling the objective of characterizing its chemical composition. The in vitro cytotoxicity and wound‐healing assays demonstrated a concentration‐dependent biological response, while the extract showed promising wound closure effects comparable to the control. Furthermore, the in vivo longevity assay using *C. elegans* supported the anti‐aging potential of the extract. Molecular docking studies revealed that the phytoconstituents, particularly caffeic acid, had notable binding affinities with MMP‐13 and GSK3‐β, aligning to explore mechanisms underlying the wound‐healing and anti‐aging effects.

## 4 Conclusion

Wound closure in all groups treated with *O. ficus‐indica* fruit juice extract (0–250 *μ*g/mL) was found to be similar to the control group. When tested for cytotoxicity, the highest survival (162.25 ± 7.48%) was observed at 250 *μ*g/mL and the lowest (111.89 ± 17.90%) at 1000 *μ*g/mL. The plant extract appears to moderately enhance the survival of *C. elegans* under heat stress, especially at 62.5 and 250 *μ*g/mL concentrations. Although statistical significance was not retained after multiple comparison corrections, the trends observed suggest a potential hormetic effect. Low‐to‐moderate doses may stimulate protective cellular mechanisms, while higher doses (e.g., 1000 *μ*g/mL) failed to provide benefit, possibly due to toxicity or reduced efficacy. These findings align with previous studies (e.g., Guerrero‐Rubio et al. 2019) and underscore the need for further research into the bioactive properties of the extract. All these findings suggest that *O. ficus‐indica* fruit juice extract, enriched with diverse secondary metabolites, could potentially be incorporated safely into wound dressings or topical applications without compromising the cell migration process, which is very important for wound healing. The fruit juice can also be consumed as a functional food for healthy aging. The wound‐healing and anti‐aging effects of the fruit juice may be due to its phenolic compounds. There is a need for further studies on its wound‐healing and anti‐aging effects. The docking study showed that the phytoconstituents of *O. ficus‐indica* fruit juice had a moderate binding potential to the target enzymes, MMP‐13 and GSK3‐β. In general, the binding potential of the phytoconstituents to MMP‐13 was higher than that of GSK3‐β. Caffeic acid gave the highest binding potential to MMP‐13 and one of the highest to GSK3‐β among the detected phytoconstituents. The study only examined a limited range of bioactive compounds, and further research could explore other components and their interactions. The long‐term effects of the juice on human health have not been investigated and should be considered in future studies. Future research should focus on further elucidating the molecular mechanisms behind the interaction of phytoconstituents with MMP‐13 and GSK3‐β, including more detailed structural studies and kinetic analyses. Additionally, exploring the long‐term therapeutic potential of *O. ficus‐indica* fruit juice in chronic wound healing and age‐related diseases could open new avenues for its clinical application.

## Author Contributions

**Gülsen Kendir:** conceptualization (equal), data curation (equal), investigation (equal), methodology (equal), resources (equal), supervision (equal), writing – original draft (equal), writing – review and editing (equal). **Meltem Güleç:** data curation (equal), formal analysis (equal), investigation (equal), software (equal), validation (equal), writing – original draft (equal). **Ayça Bal Öztürk:** data curation (equal), methodology (equal), software (equal), writing – original draft (equal), writing – review and editing (equal). **Gülşah Torkay:** formal analysis (equal), investigation (equal), validation (equal). **Muhammed Tilahun Muhammed:** formal analysis (equal), methodology (equal), software (equal), visualization (equal), writing – original draft (equal), writing – review and editing (equal). **Abdullah Olgun:** data curation (equal), methodology (equal), writing – review and editing (equal). **Ayşegül Köroğlu:** conceptualization (equal), supervision (equal), writing – review and editing (equal).

## Conflicts of Interest

The authors declare no conflicts of interest.

## Supporting information


**TABLE S1.** *Caenorhabditis elegans* thermotolerance assay.
**TABLE S2.** Calibration values for the standards.

## Data Availability

The data that support the findings of this study are available on request from the corresponding author. The data are not publicly available due to privacy or ethical restrictions.
