# Supplementary material for: Biological and Phytochemical Insights Into Opuntia ficus‐indica (L.) Mill: Cytotoxic, Wound‐Healing, and Anti‐Aging Potentials
Source: Food Sci Nutr. 2025 Jun 17;13(6):e70399. doi: 10.1002/fsn3.70399 (PMC12171788; doi:10.1002/fsn3.70399)
Supplement: Supplementary file 1 — TABLE S1. Caenorhabditis elegans thermotolerance assay. TABLE S2. Calibration values for the standards. [file FSN3-13-e70399-s001.docx]

**SUPPLEMENTARY TABLE 1** *Caenorhabditis elegans* thermotolerance assay

| Groups | Extract concentration (final, in LB with bacteria) | Number of worms |
| --- | --- | --- |
| Group 1 | 1000 µg/mL | 69 |
| Group 2 | 500 µg/mL | 89 |
| Group 3 | 250 µg/mL | 66 |
| Group 4 | 125 µg/mL | 64 |
| Group 5 | 62.50 µg/mL | 49 |
| Group 6 | 31.25 µg/mL | 71 |
| Control | 0 µg/mL | 73 |

**SUPPLEMENTARY TABLE 2** Calibration values for the standards

| Compound | Equation | Correlation | LOD (µg/mL) | LOQ (µg/mL) |
| --- | --- | --- | --- | --- |
| Caffeic acid | y= 123007.30 x -2735.42 | 0. 99875 | 0.08 | 0.25 |
| Ferulic acid | y= 98903.10 x -268.56 | 0.99629 | 0.09 | 0.28 |
| Syringic acid | y= 113296.82 x -1543.29 | 0.99701 | 0.11 | 0.34 |
| Cinnamic acid | y= 405210.5 x + 15518.96 | 0.99572 | 0.02 | 0.05 |
